# P-912. A 10-Year Retrospective, Single-Center Analysis of Nocardiosis in Immunocompetent and Immunocompromised Hosts: Clinical and Microbiological Features, Treatment Outcomes, and Determinants of Central Nervous System Involvement

**DOI:** 10.1093/ofid/ofae631.1103

**Published:** 2025-01-29

**Authors:** Francisco J Machiavello Roman, Allison Grubman, Marwan M Azar, Shelli F Farhadian

**Affiliations:** Yale School of Medicine, New Haven, Connecticut; Yale School of Medicine, New Haven, Connecticut; Yale University, New Haven, Connecticut; Yale School of Medicine, New Haven, Connecticut

## Abstract

**Background:**

*Nocardia* species are organisms with the potential to affect numerous organ systems, including the brain. However, it is unknown whether specific host or microbe factors are associated with central nervous system (CNS) nocardiosis and whether outcomes for CNS nocardiosis differ according to host immune status.

**Figure:**
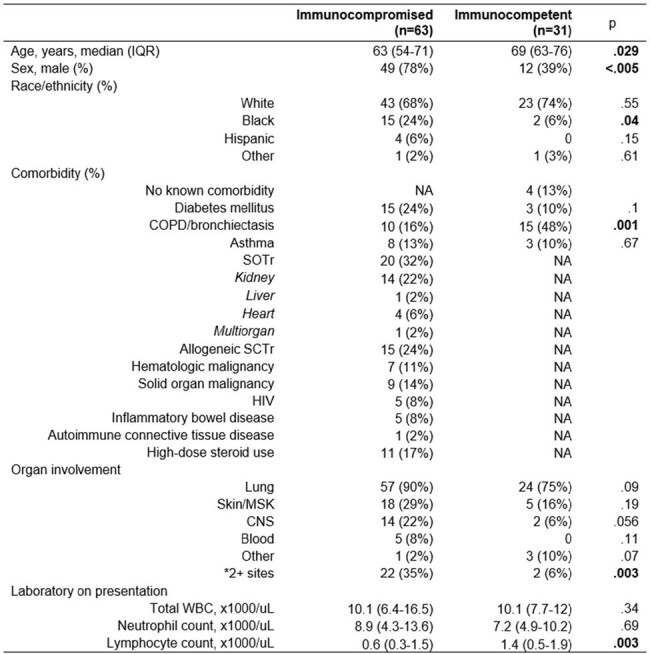

Demographics and clinical characteristics by immune status. SOTr: solid organ transplant recipient. SCTr: stem cell transplant recipient. MSK: musculoskeletal structure, such as bone or joint. COPD: chronic obstructive pulmonary disease. WBC: white blood cell count. *The isolation of Nocardia in 2 or more non-contiguous body sites constituted disseminated disease

**Methods:**

We performed a retrospective review of all adults admitted to Yale New Haven Hospital with laboratory-confirmed nocardiosis from 2013 to 2023. CNS involvement was defined as the identification of Nocardia spp on brain tissue or new radiological brain lesions in a patient with extra-neural nocardiosis. We performed multivariate logistic regression analyses of clinical and microbiological variables related to CNS involvement.

**Figure:**
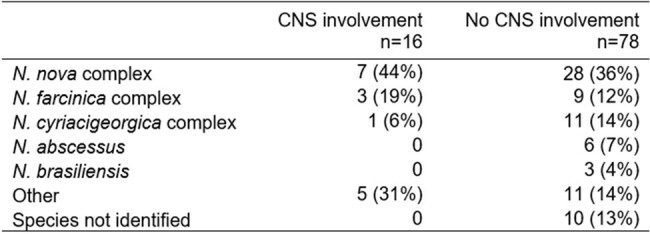

Nocardia species distribution by CNS involvement

**Results:**

94 patients were included, of which 63 (67%) were immunocompromised (Table 1). The most common immunocompromising conditions were recipients of solid organ transplants (32%) and stem cell transplants (22%). Sixteen (17%) patients had CNS involvement of their nocardiosis; this included 22% of immunocompromised and 6% of immunocompetent patients (p=.056). The most common species isolated was the *Nocardia nova* complex (Table 2). Table 3 summarizes the univariate analysis of factors associated with CNS involvement. Black race [OR 3.7 (1.2-11.7), p=.03] and *N. farcinica* infection [OR 5.9 (1.8-19.3), p=.005] were associated with increased risk of CNS involvement. In a multivariate analysis, infection by *N. farcinica* was an independent risk factor for CNS involvement [OR 5.3 (1.5-18.7), p=.009]. There were no significant survival differences in immunocompromised hosts, with or without CNS disease, compared to immunocompetent hosts.

**Figure:**
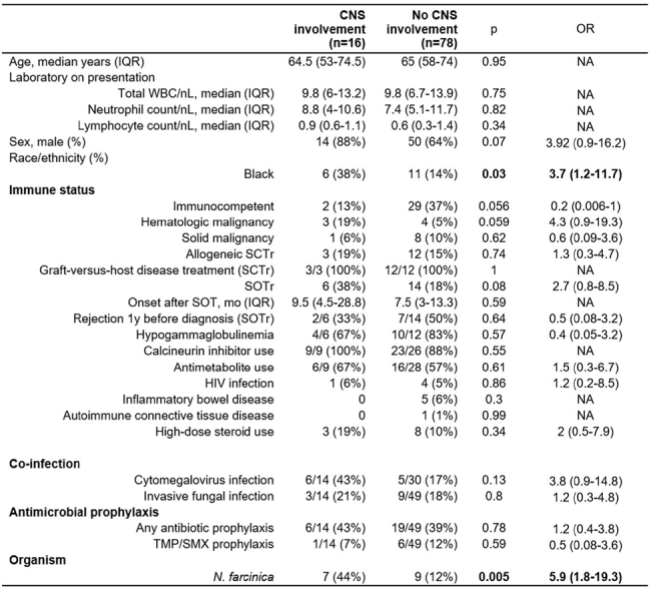

Univariate analysis of demographic, host immune status and organism features associated with CNS involvement.

**Conclusion:**

*N. farcinica* infection was identified as an independent risk factor of CNS involvement in patients with nocardiosis. Other factors related to the net state of immunosuppression were not associated with an increased risk for CNS involvement.

**Disclosures:**

**All Authors**: No reported disclosures

